# Psychometric comparison of three behavioural scales for the assessment of pain in critically ill patients unable to self-report

**DOI:** 10.1186/cc14000

**Published:** 2014-07-25

**Authors:** Gerald Chanques, Anne Pohlman, John P Kress, Nicolas Molinari, Audrey de Jong, Samir Jaber, Jesse B Hall

**Affiliations:** Department of Medicine, Section of Pulmonary and Critical Care, University of Chicago, 5841 S. Maryland Avenue MC 6076, Chicago, IL 60637 USA; Department of Anaesthesia and Critical Care Medicine, University of Montpellier Saint Eloi Hospital, 80, Avenue Augustin Fliche, 34295 Montpellier, France; Unité U1046 de l’Institut National de la Santé et de la Recherche Médicale (INSERM), Université de Montpellier 1, Université de Montpellier 2, 34295 Montpellier, France; Department of Statistics, University of Montpellier Hospitals, 371, Avenue du Doyen Gaston Giraud, 34295 Montpellier, France

## Abstract

**Introduction:**

Pain assessment is associated with important outcomes in ICU patients but remains challenging, particularly in non-communicative patients. Use of a reliable tool is paramount to allow any implementation of sedation/analgesia protocols in a multidisciplinary team. This study compared psychometric properties (inter-rater agreement primarily; validity, responsiveness and feasibility secondarily) of three pain scales: Behavioural Pain Scale (BPS/BPS-NI, that is BPS for Non-Intubated patients), Critical Care Pain Observation Tool (CPOT) and Non-verbal Pain Scale (NVPS), the pain tool routinely used in this 16-bed medical ICU.

**Methods:**

Pain was assessed by at least one of four investigators and one of the 20 bedside nurses before, during and 10 minutes after routine care procedures in non-comatose patients (Richmond Agitation Sedation Scale ≥ -3) who were unable to self-report their pain intensity. The Confusion Assessment Method for the ICU was used to assess delirium. Non-parametric tests were used for statistical analysis. Quantitative data are presented as median (25^th^ to 75^th^).

**Results:**

A total of 258 paired assessments of pain were performed in 30 patients (43% lightly sedated, 57% with delirium, 63% mechanically ventilated). All three scales demonstrated good psychometric properties. However, BPS and CPOT exhibited the best inter-rater reliability (weighted-κ 0.81 for BPS and CPOT) and the best internal consistency (Cronbach-α 0.80 for BPS, 0.81 for CPOT), which were higher than for NVPS (weighted-κ 0.71, *P* <0.05; Cronbach-α 0.76, *P* <0.01). Responsiveness was significantly higher for BPS compared to CPOT and for CPOT compared to NVPS. For feasibility, BPS was rated as the easiest scale to remember but there was no significant difference in regards to users’ preference.

**Conclusions:**

BPS and CPOT demonstrate similar psychometric properties in non-communicative intubated and non-intubated ICU patients.

**Electronic supplementary material:**

The online version of this article (doi:10.1186/cc14000) contains supplementary material, which is available to authorized users.

## Introduction

Pain is a frequent event in Intensive Care Unit (ICU) patients, with an incidence of up to 50% in medical as well as surgical patients [1–3]. Pain is associated with an acute stress response including changes in neurovegetative system activity [4], neuroendocrine secretion [5, 6] and psychological distress often manifested as agitation [7]. Improved pain management is associated with better patient outcomes in the ICU [1, 8–10]. However, pain remains currently underevaluated and undertreated [3, 11–14]. This relates to pain management being challenging in the ICU setting, particularly in patients unable to readily communicate their pain intensity, such as sedated patients and patients with delirium [15]. These patients share the common feature of a cognitive dysfunction marked by an impaired level of vigilance. Several behavioural pain scales have been developed in order to standardise the assessment of pain by healthcare providers in those non-communicative patients. The recent Clinical Practice Guidelines for the Management of Pain, Agitation, and Delirium in Adult Patients in the Intensive Care Unit [16] stated that both the Behavioural Pain Scale (BPS) [17] and the Critical Care Pain Observation Tool (CPOT) [18] demonstrated sufficient validity and reliability. However, these scales have never been compared to each other. Thus, we conducted a study in a medical ICU aimed at comparing the psychometric properties of the BPS and CPOT, as well as the Non-verbal Pain Scale (NVPS) [19, 20], which is the usual behavioural pain tool routinely used by nurses at the host institution. Because inter-rater agreement of a pain tool is paramount regarding the necessity to standardise the recognition and treatment of pain by multiple caregivers in complex non-communicative patients, our primary hypothesis was that one pain tool would be superior to others with regard to inter-rater agreement. Secondary endpoints were to evaluate validity, responsiveness and users’ preference of each tool.

## Materials and methods

### Ethics approval

The protocol was approved by the Institutional Review Board of University of Chicago Hospitals (IRB # 11-0691; Protocol Version: 7 November, 2011; Consent Version: 1 December, 2011). Written consent was obtained from the legally authorized representative or a proxy/surrogate decision-maker (patient’s next of kin) who gave consent on the patient’s behalf.

### Patient population

The study took place in the 16-bed medical ICU of the University of Chicago Hospitals, an academic tertiary care hospital, from January 2012 to June 2012 (six months). All consecutive patients ≥18 yrs old were eligible for enrolment if they had a Richmond Agitation Sedation Scale (RASS) [21, 22] above -4 and were unable to self-rate their pain intensity with the Visually Enlarged 0 to 10 Numeric Rating Scale (0 to 10 V-NRS). This scale is adapted to ICU patients and demonstrated to be the most feasible self-report pain scale in the ICU setting [23]. Exclusion criteria were neurological disorder, decision to withdraw life-support or unstable condition preventing planned routine care procedures.

### Conduct of the study

Investigators screened patients daily for eligibility including RASS assessment, self-report pain ability by the patient and possibilities to plan any routine procedures of care with the bedside nurse. After having obtained consent from the surrogate decision-maker and having enrolled the patient into the study, investigators planned different procedures of care with the bedside nurse including: (1) a simple repositioning of the patient in the bed (moving the patient up or onto their side), (2) a complete turning of the patient onto both sides in order to wash their back and change the sheets, (3) a tracheal suctioning if possible (intubated patients), and (4) a mobilisation by physiotherapist/occupational therapist if possible.

### Data handling

#### Pain

Pain evaluation using the three different behavioural pain tools (BPS, CPOT, NVPS) was independently performed at the same time by two or three paired evaluators (one or two investigators, and the bedside nurse) in three conditions for each patient: (1) at rest, before any procedure; (2) during the care procedure; and (3) 10 minutes after the procedure. Every patient was assessed during a simple repositioning and a complete turning on both sides. Patients were evaluated during tracheal suctioning or mobilisation if possible. Turning and suctioning were chosen because they are the most common and/or painful procedures in the ICU setting [24, 25]. Repositioning, turning and mobilisation were chosen so that different intensities of stimulation could be compared to each other.

For all these measurements, investigators and the bedside nurse were blinded to each other, each observer using a separate sheet (see Additional file 1). Scale order was determined by randomisation software and printed as a list of combinations before the beginning of the study. Order of occurrence of a given scale was tested to assure that no scale would have a preferred order of occurrence. The randomisation of scale order was considered as a gold standard to take into account any learning effect or, on the contrary, any fatigability during a study procedure incorporating several pain tools [26]. The nurse manager and the investigator team informed the bedside nurses about the study purposes before the study began. Moreover, pain tools descriptors and instruction for use were explained to the bedside nurses by the investigator team before the first procedure for each patient. Published educational tools for BPS/BPS-NI [27] and CPOT [28], as well as the most recent revised version of the NVPS [20], were used for this educational purpose in the determined randomised order. Content details of the three tools are given in the additional file (see Additional file 1). All observers had to rate every domain of the pain tools on a sheet where descriptors of the tools were written to avoid any learning issues (see Additional file 1). A simplified comparison of the three tools structure is shown in Table 1. Each of the three tools requires observing three different kinds of behavioural domain related to pain: patient’s face, muscular movements and/or tonus, breathing and/or vocalisation. In addition, NVPS requires observing physiological signs (Table 1).

**Table 1:** **Structure comparison of the three behavioural pain tools**

BPS	CPOT	NVPS
Number of observation domains	Number of observation domains	Number of observation domains
3	4	6
Number of descriptors per domain	Number of descriptors per domain	Number of descriptors per domain
4 (rated 1 to 4)	3 (rated 0 to 2)	3 (rated 0 to 2)
Total score 3 - 12	Total score 0 - 8	Total score 0 - 12
**Facial domains**		
Face	Face	Face
**Breathing domains**		
Mechanical ventilation or vocalisation	Mechanical ventilation or vocalisation	Respiration
**Muscular domains**		
Upper limbs movements	Body movements	Activity
	Muscle tension	Guarding
**Physiological domains**		
		Physiological I (vital signs)
		Physiological II (skin and pupils)

BPS, Behavioral Pain Scale; CPOT, Critical-Care Pain Observation Tool; NVPS, Non-verbal Pain Scale.

Throughout the manuscript, we use the word BPS that includes both BPS and its adaptation for non-intubated patients (BPS-NI), similarly to the CPOT that includes both types of descriptors, either for intubated or non-intubated patients.

### Demographic and medical data

Age, gender, height and weight, co-morbidities, and reason for admission to the ICU were recorded. Acute Physiology and Chronic Health Evaluation (APACHE) II score and Sequential Organ Failure Assessment (SOFA) score [29] were calculated within 24 hours after ICU admission and before enrolment, respectively. Body mass index (BMI) was calculated as the ratio (kg/m^2^) between weight (kg) and height squared (m^2^). Type and doses of sedatives and analgesic drugs were collected before any procedures. In addition to the RASS measurement by investigators, delirium was assessed upon enrolment by the Confusion Assessment Method for the ICU (CAM-ICU) [30, 31]. Physiological parameters (heart and respiratory rates, systolic, diastolic and mean arterial blood pressure, pulse oximetry) were continuously measured through bedside monitoring and retrospectively recorded by investigators to fit with the NVPS description [20].

### Statistical analysis

#### Measurement of psychometric properties

Psychometric properties related to the use of pain tools were assessed using the new terminology [32] as recommended by recent Clinical Practice Guidelines for the Management of Pain, Agitation, and Delirium in Adult Patients in the Intensive Care Unit [16].

1.1 *Inter-rater reliability*
  Inter-rater reliability of the three tools (primary endpoint) was tested by the weighted kappa coefficient. A kappa coefficient above 0.80, 0.60 and 0.40 is considered as measuring respectively a ‘near perfect’, ‘important’ and ‘moderate’ agreement [33]. Comparisons of kappa coefficients between scales were made using the z test [34].
  To deal with repeated measurements, a sensitivity analysis was performed taking into account first assessments only, as previously described [22]. Moreover, the inter-rater agreement within an error of one mark was calculated as the ratio, expressed in percentage, between the number of scores obtained with each scale that differed by not more than one point between different observers, and the total number of scores. Comparisons between scales were made using chi-square test.
1.2 *Internal consistency*
  Internal consistency was measured using the Cronbach-α method [35]. A Cronbach-α value higher than 0.7 reflects a satisfactory internal consistency, that is a high inter-relation between each domain of the tool [35]. Cronbach-α coefficients were compared between the three scales using the method by Feldt [36].
1.3 *Discriminant validation*
  Discriminant validation was determined by comparing total scores obtained during different situations and stimuli, that is at rest and during a procedure (suctioning, repositioning or turning) as well as during procedures with different durations and intensities, that is during a simple repositioning and during a complete turning. The Mann-Whitney-Wilcoxon test was used to test the difference between two different situations. We tested the responsiveness of the three tools as another way to measure change, that is the ability to detect change regarding different situations even if those changes are small. The magnitude of this property was assessed by the effect size [37]. The effect size coefficient is considered small if it is less than 0.20, moderate if it is near 0.50, and large if it is more than 0.80 [37]. The modified Jackknife method was used to test any significant difference in responsiveness between two scales [38].
1.4 *Feasibility*
  Feasibility was assessed by administering a standardised questionnaire once to the bedside nurses during their initial participation in the study interventions. The nurses were asked to rate their preference of each particular pain scale, as well as the degree of accuracy when used for routine practice or research purposes, and the ease of learning.

#### Primary endpoint and power analysis

The primary endpoint was the inter-rater reliability because this psychometric property is paramount and, if deficient, precludes implementation of a pain tool and associated diagnostic and therapeutic pain strategies by the ICU team [1, 4, 16]. The number of paired assessments (assessment by investigators + assessment by the ICU clinical staff) needed to show a weighted kappa difference of 0.1 from a given kappa of 0.80 (±0.10), with an α of 0.05 and a β of 0.20, was determined to be n = 167 paired assessments. Considering that post-procedure assessments might not be different than pre-procedure assessment, only the pre- and per-procedure assessments were included, that is at least 85 paired assessments before and 85 paired assessments during the procedure, which is equal to 170 paired assessments. Because each patient could be assessed during two to three procedures by two to three observers, the number of patients necessary to enrol was n = 30 to reach these 170 paired assessments.

#### Presentation of data

Quantitative data are shown as medians and 25^th^ to 75^th^ percentiles. A *P* value of ≤0.05 was considered statistically significant. Data were analysed using the SAS software version 9.1 (SAS Institute, Cary, NC, USA).

## Results

**Figure 1:**
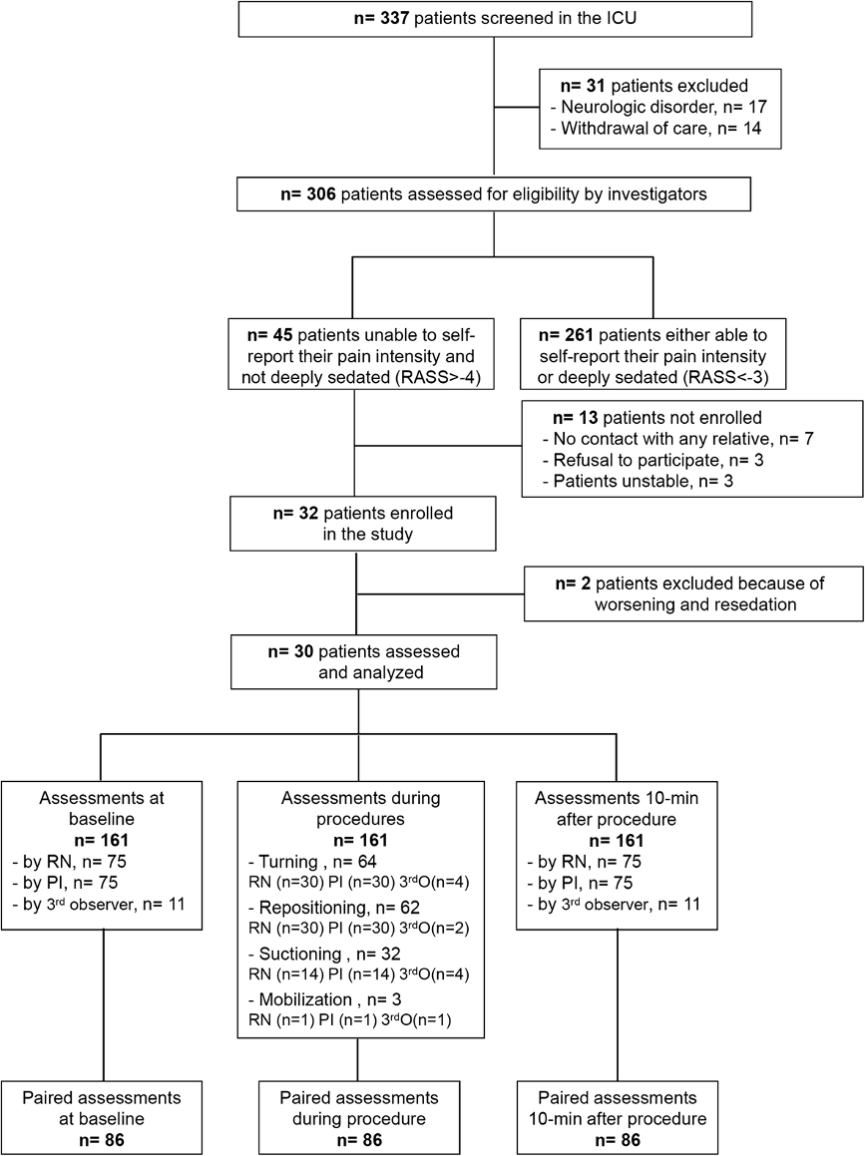
**Study flow chart.**

During the study period, 258 paired observations of pain behaviour were done with each pain tool in 30 patients by 24 observers (20 registered nurses (RNs), 4 investigators) during 75 procedures: repositioning, n = 30; turning onto both sides for bathing, massage and changing the sheets, n = 30; suctioning, n = 14; mobilisation for physical therapy, n = 1. A consort flow chart of patient enrolment is shown in Figure 1. Table 2 summarises patients’ demographic and medical characteristics.

**Table 2:** **Demographic and medical characteristics of the 30 patients included for analysis**

Age (years)	67 [57-74]
Sex (F/M)	19/11
Body mass index (kg/m^-2^)	26 [22-30]
Chronic pain syndrome, n (%)	11 (36%)
Reason for admission to the ICU	
*Acute respiratory failure, n (%)*	17 (57%)
*Severe sepsis/septic shock, n (%)*	8 (27%)
Miscellaneous*, n (%)	6 (20%)
Time between admission to ICU and enrolment (days)	4 [2-7]
APACHE II score within 24 h after admission to ICU	23 [20-29]
SOFA score upon enrolment	8 [7-11]
Mechanical ventilation upon enrolment, n (%)	19 (63%)
Sedation upon enrolment	13 (43%)
*Propofol, n (%)*	*12 (40%)*
*Dose (μg.kg* ^*-1*^ *.min* ^*-1*^ *)*	*10* [5-11]
*Dexmedetomidine, n (%)*	*1 (3%)*
Analgesia upon enrolment	16 (53%)
*Fentanyl, n (%)*	*15 (50%)*
*Dose (μg.kg* ^*-1*^ *.h* ^*-1*^ *)*	*0.9 [0.6-1.2]*
*Hydromorphone, n (%)*	*1 (3%)*
RASS level	-1 [-3; +1]
*RASS level = 0, n (%)*	*4 (13%)*
*RASS level >0, n (%)*	*6 (20%)*
*RASS level <0, n (%)*	*20 (67%)*
CAM-ICU positive in non-sedated patients, n/N (%)	17/17 (100%)

Continuous data are expressed in median [25^th^ to 75^th^ percentiles]. *Miscellaneous reasons for admission to the ICU: metabolic, acute hepatitis, altered mental status, mechanical ventilation weaning, agitation post procedure.

ICU, Intensive Care Unit; APACHE II score, Acute Physiology And Chronic Health Evaluation II score; SOFA, Sequential Organ Failure Assessment; RASS, Richmond Agitation Sedation Scale; CAM-ICU, Confusion Assessment Method for the Intensive Care Unit.

### Inter-rater reliability (primary endpoint)

Inter-rater reliability was evaluated by weighted kappa coefficients, which are summarised in Table 3. The reliability was nearly perfect for BPS and CPOT and important for NVPS. Weighted kappa coefficients were significantly greater for BPS (0.81 ± 0.03) and CPOT (0.81 ± 0.03) than for NVPS (0.71 ± 0.04, *P*<0.05 compared to BPS and CPOT). Using only the first assessments for each patient, the weighted kappa coefficients for BPS, CPOT and NVPS were unchanged at 0.88, 0.80 and 0.67, respectively.

**Table 3:** **Inter-observer reliability measured by weighted kappa coefficients for each of the three pain tools**

BPS	CPOT	NVPS
Total score	Total score	Total score
0.81 (0.03)^a^	0.81 (0.03)^a^	0.71 (0.04)
Facial domains		
Face	Face	Face
0.75 (0.03)	0.81 (0.03)^a,c^	0.70 (0.04)^d^
Breathing domains		
Ventilation/vocalisation	Ventilation/vocalisation	Respiration
0.78 (0.04)^a^	0.71 (0.05)^a,c^	0.54 (0.07)^e^
Muscular domains		
Upper limbs	Body movements	Activity
0.61 (0.06)	0.42 (0.07)^b^	0.52 (0.06)
	Muscle tension	Guarding
	0.43 (0.07)^b^	0.32 (0.07)^b^
Physiological domains		
		Physiological I
		0.46 (0.08)
		Physiological II
		0.02 (0.03)^f^

All data are expressed in weighted kappa coefficient (standard deviation). ^a^ *P*<0.05 compared to NVPS; ^b^ *P*<0.05 compared to BPS; ^c^ *P*<0.05 compared to CPOT muscular domains; ^d^ *P*<0.05 compared to NVPS non-facial domains; ^e^ *P*<0.05 compared to NVPS guarding; ^f^ *P*<0.05 compared to NVPS non-physiological II domains. BPS, Behavioral Pain Scale; CPOT, Critical-Care Pain Observation Tool; NVPS, Non-verbal Pain Scale.

Table 3 shows inter-rater reliability for each tool’s domain. For the facial domain, the greater reliability was demonstrated for CPOT, which was significantly greater than NVPS. For the muscular domains, the greater reliability was demonstrated for BPS, which was significantly greater than the two muscular domains of the CPOT and one of the NVPS muscular domains (Table 3). The three domains of the BPS demonstrated similar reliability. For the CPOT, both facial and breathing domains demonstrated a significantly greater reliability than muscular domains. For the NVPS, the facial domain demonstrated a significantly greater reliability than other domains. Apart from the facial domain, the breathing domain of the NVPS demonstrated the greater reliability and the physiological domain II the lowest. A subgroup analysis was performed on patients according to their intubation status. In intubated and non-intubated patients, BPS and CPOT had the highest inter-rater reliability but the difference was only significant between BPS and NVPS in non-intubated patients (0.89 ± 0.04 vs. 0.74 ± 0.05, *P*<0.05). Inter-rater reliability was not significantly different in intubated compared to non-intubated patients for NVPS (0.71 ± 0.04 vs. 0.74 ± 0.05) and CPOT (0.80 ± 0.03 vs. 0.82 ± 0.05). BPS had a significantly greater inter-rater reliability in non-intubated than intubated patients (0.89 ± 0.04 vs. 0.77 ± 0.04, *P*<0.05). Finally, within an error of one point, inter-rater agreement was significantly (*P*<0.01) greater for BPS (81%) and CPOT (77%) than for NVPS (65%) for all the observations (before and during the procedures), as well as for observations made during the procedures only (BPS, 73%; CPOT, 77%; NVPS, 57%; *P*<0.05 between NVPS and the two other scales).

### Internal consistency

Measurement of Cronbach-α coefficients showed a satisfactory internal consistency for each of the three scales: 0.80 for BPS, 0.81 for CPOT and 0.76 for NVPS. Cronbach-α was significantly greater for BPS (*P*<0.01) and CPOT (*P*<0.001) compared to NVPS. The difference between BPS and CPOT was not significantly different (*P* = 0.48).

There was no significant difference in Cronbach-α coefficients between intubated and non-intubated patients for BPS (0.81 for intubated patients and 0.83 for non-intubated patients, *P* = 0.15) and CPOT (0.82 for intubated patients and 0.81 for non-intubated patients, *P* = 0.99) contrary to NVPS (0.79 for intubated patients and 0.46 for non-intubated patients, *P* <0.001).

### Discriminant validation

**Figure 2:**
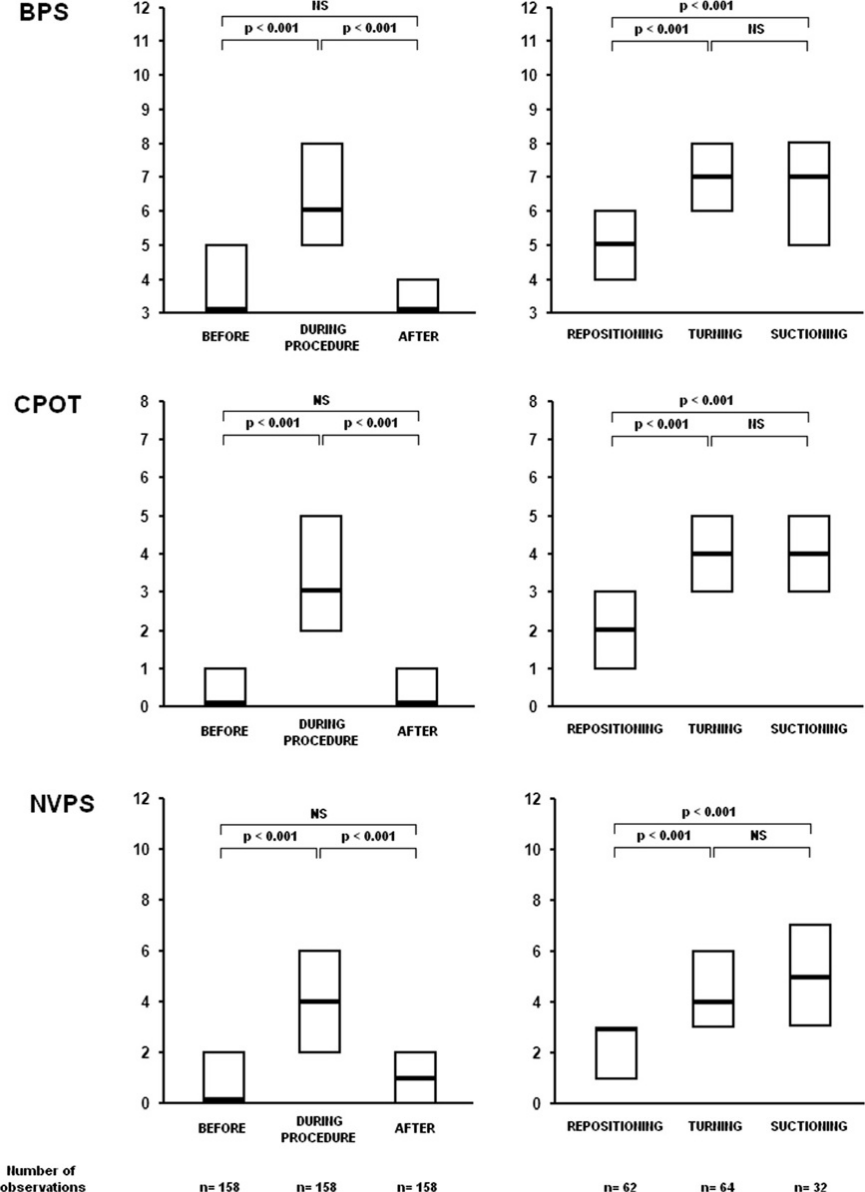
**Median scores observed by all the observers with each of the three tools, according to different situations.** This figure shows the median scores of the three tools evaluated by all the observers according to different situations: before, during and after repositioning, turning and suctioning. The left figures show that there was a significant increase in each of the three scores from baseline to procedure and a significant decrease 10 minutes after the procedure. The right figures showed the scores measured during the different procedures. Among them, turning and suctioning were significantly the most painful.

Figure 2 shows the median scores of the three tools evaluated by all the observers according to different situations. There was a significant increase in each of the three scores from baseline to procedure (*P*<0.001) and a significant decrease 10 minutes after the procedure (*P*<0.001). The median scores were not significantly different between observations made at baseline and observations made after the procedure (BPS, *P* = 0.41: CPOT, *P* = 0.74; NVPS, *P* = 0.89). Discriminant validation was also tested comparing median scores observed during two similar situations differing by the intensity and the length of the procedures, that is repositioning and turning onto both sides. There was also a significant difference between these two procedures for each of the three tools (*P*<0.001). Finally, turning and suctioning were the most painful procedures (Figure 2). Difference of pain scores between these two procedures was not significant (BPS, *P* = 0.90: CPOT, *P* = 0.68; NVPS, *P* = 0.40).

Responsiveness of the scales was tested by the effect size coefficient, which was large (>0.80) for each of the three scales when calculated between baseline and observations done during the procedures: BPS = 1.99; CPOT = 1.55; NVPS = 1.46. BPS and CPOT demonstrated a significantly higher responsiveness than NVPS, as well as BPS compared to CPOT. The effect size coefficients also remained large when calculated between the repositioning and turning procedures (BPS = 0.90; CPOT = 0.86; NVPS = 0.92), without any significant differences between the three scales.

### Feasibility

**Figure 3:**
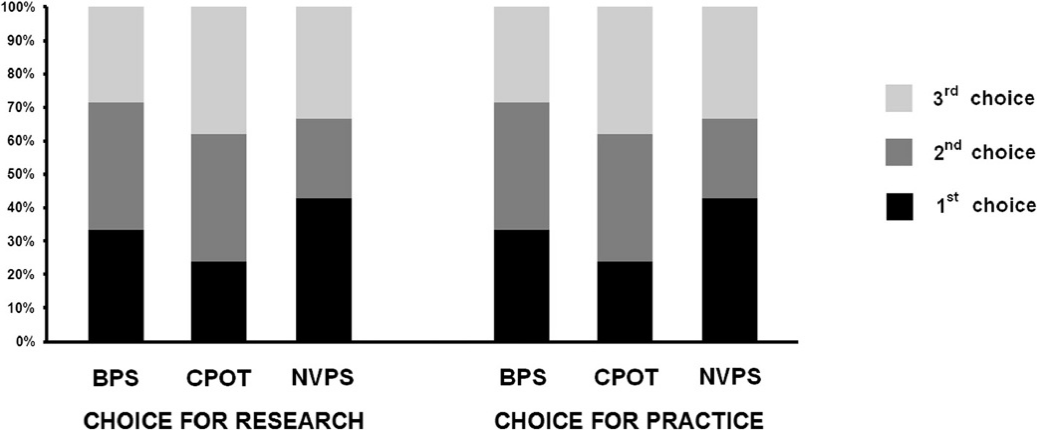
**Preference about the use of the three tools, rated by the 20 nurses and the nurse manager.** This figure shows that NVPS was the preferred tool, following by the BPS but the difference was not significant compared to the others (*P* = 0.68 for research and for practice). BPS, Behavioral Pain Scale; NVPS, Non-verbal Pain Scale.

The 20 RNs who participated in the study and the nurse manager (one of the investigators) rated the three tools at a median of 7 to 8 (0 = the worst, 10 = the best) for accuracy, usefulness and ease of learning. The BPS was rated higher with regard to ease of learning than the CPOT (*P* = 0.02), but the BPS was the same as the NVPS (*P* = 0.07): BPS, 8 [7–10]; CPOT 8 [5–8], NVPS 8 [6–8]. There was no significant difference (all *P* values >0.49) between the three tools either with regard to accuracy (BPS, 7 [7, 8]; CPOT 8 [5–8], NVPS 7 [6–8]) or usefulness (BPS, 7 [5–8]; CPOT 8 [5–8], NVPS 7 [6–8]). Observers’ preference for the three tools is shown in Figure 3. There was no difference between preference of use either for research or routine practice. The NVPS was chosen as the preferred tool the most often (43%), followed by the BPS (33%) and the CPOT (24%), but the difference was not significant. Among the nine observers who chose the NVPS as the preferred tool, four explained their choice resulting from their being more familiar with the scale. Reasons for preferential choice are given in Table 4. Most of the arguments were given by some observers as positive (explaining their first choice) but also by other observers as negative (explaining their last choice).

**Table 4:** **Reasons of preferred tool choice by the 20 nurses and the nurse manager**

	Reasons given for first choice	Reasons given for last choice
**BPS**	n = 7	n = 6
	Main reasons:	Main reasons:
	Simplicity, easiness, n = 4	Simplicity, n = 1
	Descriptors clear or precise, n = 2	Descriptors less well described, n = 1
	4 descriptors instead of 3, n = 1	Less specific, n = 1
		Less information, n = 3
**CPOT**	n = 5	n = 8
	Main reasons:	Main reasons:
	Descriptors more detailed, n = 2	Descriptors too complex, n = 2
	Descriptors better described, n = 2	Descriptors less well detailed or confusing, n = 3
	Vocalisation domain compared to NVPS, n = 1	No reason, n = 3
	*Other reason:*	
	*Ventilator alarm notified, n = 1*	
**NVPS**	n = 9	n = 7
	Main reasons:	Main reasons:
	Familiar with, n = 4	Some descriptor not understandable, n = 1
	More information, n = 3	Descriptors less well detailed, n = 2
	Vital signs notified, n = 2	Vital signs not valid in ICU patients, n = 3
		No reason, n = 1
	*Other reasons:*	
	*Vital signs notified, n = 1*	
	*Change over time notified, n = 1*	

BPS, Behavioral Pain Scale; CPOT, Critical-Care Pain Observation Tool; NVPS, Non-verbal Pain Scale.

## Discussion

The main findings of this study are that BPS, CPOT and NVPS have good psychometric properties but BPS and CPOT have significantly higher inter-rater reliability, internal consistency and responsiveness than NVPS. Discriminant validation was good for all three scales. There was no difference in regards to feasibility except for BPS, which is rated a little easier to remember than the other scales, with only three domains of observation rather than four and six for CPOT and NVPS. Scales’ preference was variable among users, with no scale demonstrating any consensus. In all, either BPS or CPOT appear to be superior tools and should be chosen in the ICU where no behavioural pain scale has been implemented yet, consistent with the recent Practice Guidelines [16].

These data are consistent with a recent study aimed at comparing CPOT and NVPS in mostly intubated patients, which found a better inter-rater reliability for CPOT [39]. Moreover, our study showed that BPS and CPOT can be used in both intubated and non-intubated patients whereas NVPS demonstrated a poor internal consistency in non-intubated patients. NVPS was neither constructed nor validated in non-intubated patients [19, 20] in contradistinction to the BPS and CPOT that are both constructed to be used either in intubated or non-intubated patients [17, 18, 27]. It could not have been possible to compare BPS and CPOT in an ICU team trained to use one of those tools. In our institution, nurses are trained to use the NVPS, which consequently allows for an accurate comparison between BPS and CPOT in a team familiar with using a behavioural pain tool. Moreover, nurses in our institution routinely use the NVPS to also assess pain in non-intubated patients unable to self-report. NVPS’ internal consistency was indeed low in non-intubated patients. However, inter-rater reliability was not significantly different for NVPS depending on whether the patients were intubated or not. The reliability of the BPS was significantly greater in non-intubated patients. BPS requires assessing ventilator waveforms and asynchrony, which could be difficult while observing patients’ face and body at the same time. Listening to ventilator alarms like for the CPOT could be a useful alternative. Recent American Practice Guidelines recommended further assessment in non-intubated patients with a modified BPS (that is BPS-NI) or the CPOT. These new data should strengthen the rationale for BPS and CPOT use in ICU non-intubated non-communicative patients.

Pain is one of the most stressful events experienced by patients during their ICU stay [40, 41]. At rest, surgical and trauma patients report surgery/trauma site as the most painful area although medical patients most likely report pain localised in back and limbs [2]. Being moved for nursing-care procedures is one of the most painful procedures experienced by the patient during the ICU stay whatever the type of admission (medical, surgical or trauma) [3, 24, 25, 42, 43]. Contrary to pain while moving the patient for nursing procedures, pain during active mobilisation for early rehabilitation had never been investigated in the ICU-setting [44] until the recent EUROPAIN™ study [25]. In this large multicentre study assessing 13 different procedures of care in ICU patients, active mobilization was the less painful procedure (NRS = 2 [0;5]) while positioning and turning were associated with a higher pain intensity (3 [0;5] and 3 [0.25;6], respectively) [25]. One of the differences between active and passive mobilization (that is rehabilitation vs. repositioning and turning) is that movements and pressure on body parts can be controlled by the patients or not. This could explain the difference in pain intensity between these procedures. However, whether pain could be a barrier toward early rehabilitation in specific ICU patients, such as surgical patients, remains unknown [45, 46]. In the present study, we were able to enrol only one patient while being mobilised by a physiotherapist/occupational therapist. This was because mobilisation requires the patient to participate and be able to follow instructions and our inclusion criteria specifically enrolled patients unable to self-report their pain intensity, a less common feature in patients able to participate in early mobility. The one patient enrolled for mobilisation in our trial was effectively with delirium and was not able to use the 0 to 10 NRS. However, early mobilisation could prevent delirium in the ICU and is therefore recommended in patients able to participate. Along with delirium, pain is one other neuropsychological event for which an accurate management is highly recommended in ICU patients. Improved pain management based on an accurate assessment of patient’s pain intensity is associated with better patient outcomes in the ICU [1, 8–10]. Sequential studies using the BPS performed in surgical and medical ICUs reported that a multidisciplinary (nurse and physician) protocol to diagnose and manage pain, agitation and delirium was associated with a reduced duration of mechanical ventilation [1, 10], ICU-acquired infections [1], length of stay in ICU and hospital as well as 30-day mortality [10]. A large multicentre observational study in 1,144 mechanically ventilated patients, in whom BPS was the most frequently used tool, showed that pain assessment was associated with reduced duration of mechanical ventilation and length of stay in ICU [9]. That could be explained in part by a reduced use of sedatives and a greater use of analgesics [9]. Implementation of the CPOT was also associated with a reduction of sedatives and change in analgesics ordering [28, 47], suggesting that standardising pain assessment in critically ill patients may allow for a better match between analgesics requirements and administration. Recently, a multidisciplinary quality-improvement study based on pain assessment using the 0 to 10 V-NRS and BPS/BPS-NI along with an analgesia protocol showed that decreased incidence in severe pain while turning ICU patients was associated with decreased adverse outcomes [4]. Therefore, pain management is highly challenging in the ICU setting and determining the most valid and reliable tool is paramount before any implementation of an analgesia protocol to a multidisciplinary team [16]. The team’s preference regarding the choice of a pain tool should also be taken into account but a consensus might be difficult to reach. Indeed, no tool reached a consensus among users in our study. One-third of users who chose NVPS as the preferred tool mentioned observation of vital signs as the reason. Inversely, almost half of the users who ranged NVPS as the less preferred tool mentioned that observation of vital signs was not accurate in critically ill patients. Indeed, the physiological domains of NVPS demonstrated poor to just moderate inter-rater reliability despite objective measurement and recording of vital signs. Because pain can be associated either with an increase or decrease in physiological variables [48], which can moreover be influenced by many factors such as disease or treatment, variation of vital signs should be studied further in critically ill patients in order to standardise them as a possible domain in observational pain tools. Another example highlighting difficulties in reaching a consensus among users is the subjective assessment of tool’s complexity. One-quarter of users found the BPS too simple or with less information whereas another quarter found the CPOT too complex or with descriptors less well detailed or confusing. However, complexity of a subjective tool may impact on inter-rater reliability. Thus, the higher reliability shown for the muscular domain of BPS compared to CPOT and NVPS might be potentially explained by the fact that both CPOT and NVPS have two muscular domains while BPS has only one.

Finally, if using tools demonstrating the best psychometric properties such as BPS or CPOT might be recommended, it is unknown whether a small but significant difference in psychometric measurement is clinically relevant or not in regard to patients’ outcome. Also, clinical studies are still needed to determine which threshold is the most effective in regard to ICU outcome (duration of mechanical ventilation, stress response-related events) but also in regard to outcome after ICU discharge (chronic pain syndrome, post-traumatic stress disorder (PTSD)). Then, further studies are needed to determine how it would be the most effective to educate, train and assess healthcare givers when using subjective behavioural pain tools to increase their reliability in research and routine use. Results of this study showed that repeated education and training is paramount to assure important inter-rater reliability of a tool as previously showed with the use of sedation and delirium tools in the ICU setting [49]. A different education strategy and/or tool training prior to the present study might have resulted in different findings. Whether some investigators who could have been more experienced about NVPS or BPS/BPS-NI use might have impacted on the results should be considered as a possible bias and a limit of the study. In order to minimize educational issues, descriptors and instructions for use were clearly indicated on the data collection sheet for the three tools (see Additional file 1). Also, this could explain that all three tools demonstrated good psychometric properties.

## Conclusions

BPS, CPOT and NVPS demonstrate good inter-rater reliability in both intubated and non-intubated ICU patients unable to self-report their pain intensity. BPS and CPOT have significantly higher inter-rater reliability, internal consistency and responsiveness than NVPS, which psychometric properties remain, however, acceptable in general but not for the physiological domains. Discriminative validation is important for all three scales. There is no difference in regard to feasibility except for BPS, which is rated a little easier to remember. However, no scale demonstrated any consensus among users. Either BPS or CPOT should be used in intubated and non-intubated patients unable to self-report, particularly when no behavioural pain scale is already available in an ICU setting.

## Key messages

- BPS and CPOT have significantly higher inter-rater reliability and internal consistency than NVPS in intubated and non-intubated ICU patients unable to self-report their pain intensity.
- BPS demonstrates significantly highest responsiveness.
- Psychometric properties are acceptable for NVPS in general but not for the physiological domains.
- No scale demonstrates a better feasibility among users.
- Because of significantly better psychometric properties, either BPS or CPOT should be used in intubated and non-intubated ICU patients unable to self-report.

## Electronic supplementary material

Additional file 1: **Data sheet for observers’ pain assessments.** This additional file provides the sheet used by the observers during the study to independently assess pain with each of the three tools: BPS, CPOT and NVPS. Note that descriptors and instruction of use were written for each tool to avoid any learning issues. (PDF 177 KB)

## Abbreviations

APACHE: Acute Physiology and Chronic Health Evaluation
BMI: body mass index
BPS: Behavioral Pain Scale
CAM: Confusion Assessment Method
CPOT: Critical-Care Pain Observation Tool
ICU: Intensive Care Unit
NRS: Numeric Rating Scale
NI: non-intubated
NVPS: Nonverbal Pain Scale
PTSD: post-traumatic stress disorder
RASS: Richmond Agitation Sedation Scale
RN: registered nurse
SAPS: Simplified Acute Physiological Score
SOFA: Sequential Organ Failure Assessment score.
