# Supplementary material for: Psychometric comparison of three behavioural scales for the assessment of pain in critically ill patients unable to self-report
Source: Crit Care. 2014 Jul 25;18(5):R160. doi: 10.1186/cc14000 (PMC4220092; doi:10.1186/cc14000)
Supplement: Supplementary file 1 — Additional file 1: Data sheet for observers’ pain assessments. This additional file provides the sheet used by the observers during the study to independently assess pain with each of the three tools: BPS, CPOT and NVPS. Note that descriptors and instruction of use were written for each tool to avoid any learning issues. (PDF 177 KB) [file 13054_2014_2900_MOESM1_ESM.pdf]

Patient sticker

Date (mm/dd/yyyy) : __/__/2012 OBSERVER NAME:_____________

Time (hh:mm) : __ : __ AM PM

Type of procedure □ turning of the side for cleaning □ repositioning

□ mobilization PT/OT □ other:…………………

	BPS At rest (before procedure)	BPS max.value during procedure	BPS At rest (10 min after procedure)
**1. Facial expression (1-4) =**			

1 Relaxed
2 Partially tightened (brow lowering)
3 Fully tightened (eyelid closing)
4 Grimacing (folded cheek)

**2. Movement of upper limbs (1-4) =**			

At rest: check tonus by mobilization of the limb

1 No movement
2 Partially bent
3 Very bent with finger flexion
4 Retracted, opposition to care

**3.a. Compliance with ventilation (1-4) =**			

1 Tolerating ventilation
2 Coughing but tolerating ventilation most of the time
3 Fighting ventilator but ventilation possible sometimes
4 Unable to control ventilation

OR

**3.b. Vocalization (1-4) =**			

(non intubated patient)

1 No pain vocalization
2 Moaning not frequent and not prolonged (<3 sec ou 3/min)
3 Moaning frequent or prolonged (>3 sec ou 3/min)
4 Howling or verbal complaints including « ow, ouch» or breath-holding

Patient sticker

Date (mm/dd/yyyy) : __/__/2012 OBSERVER NAME:___________
Time (hh:mm) : __ : __ AM PM
Type of procedure □ turning of the side for cleaning □ repositioning
□ mobilization PT/OT □ other:…………………

	**CPOT At rest (before procedure)**	**CPOT max.value during procedure**	**CPOT At rest (10 min after procedure)**
**1. Facial expressions (0-2) =**			

0 Relaxed, neutral : No muscle tension observed
1 Tense : Presence of frowning, brow lowering, orbit tightening and levator contraction or any other change (e.g. opening eyes or earing during nociceptive procedures)
2 Grimacing : All previous facial movements plus eyelid tightly closed : the patient may present with mouth open or biting the endotracheal tube

**2. Body movements (0-2) =**			

0 Absence of movement or normal position : Does not move at all (doesn't necessarily mean absence of pain) or normal position (movements not aimed toward the pain site or not made for the purpose of protection)
1 Protection : Slow, cautious movements, touching or rubbing the pain site, seeking attention through movements
2 Restlessness : Pulling tube, attempting to sit up, moving limbs/thrashing, not following commands, striking at staff, trying to climb out of bed

**3.a. Compliance with the ventilator (0-2) =**			

0 Tolerating ventilator or movement: Alarms not activated, easy ventilation
1 Coughing but tolerating: Coughing, alarms may be activated but stop spontaneously
2 Fighting ventilator: Asynchrony: blocking ventilation, alarms frequently activated

OR

**3.b. Vocalization (0-2) =** *(non intubated patient)*			

0 Talking in normal tone or no sound: Talking in normal tone or no sound
1 Sighing, moaning: Sighing, moaning
2 Crying out, sobbing: Crying out, sobbing

**4.Muscle tension** (0-2)			

Evaluation by passive flexion and extension of upper limbs when patient is at rest or evaluation when patient is being turned

0 Relaxed: No resistance to passive movements
1 Tense, rigid: Resistance to passive movements
2 Very tense or rigid: Strong resistance to passive movements, incapacity to complete them

Patient sticker

Date (mm/dd/yyyy) : __/__/2012 OBSERVER NAME:____________

Time (hh:mm) : __ : __ AM PM

Type of procedure □ turning of the side for cleaning □ repositioning

□ mobilization PT/OT □ other:…………………

	NVPS At rest (before procedure)	NVPS max.value during procedure	NVPS At rest (10 min after procedure)
**1. Face (0-2) =**			
**2. Activity (movement) (0-2) =**			
**3 Guarding (0-2) =**			
**4 Physiologic I (vital signs) (0-2) =**			
**5.a Respiratory** (0-2)			
**5.b PhysiologicII (0-2) =**			

1. Face (0-2) =

0 No particular expression or smile
1 Occasional grimace, tearing, frown or wrinkled forehead
2 Frequent grimace, tearing, frown or wrinkled forehead

2. Activity (movement) (0-2) =

0 Lying quietly, normal position
1 Seeking attention through movement of slow cautious movements
2 Restless activity and/or withdrawal reflexes

3 Guarding (0-2) =

0 Lying quietly, no positioning of hands over areas of body
1 Splinting areas of the body, tense
2 Rigid, stiff

4 Physiologic I (vital signs) (0-2) =

0 Stable vital signs, no change in past 4 hours
1 Change over past 4 hours in any of the following: SBP >20 HR >20 RR >10
2 Change over past 4 hours in any of the following: SBP >30 HR >25 RR >20

5.a Respiratory (0-2)

0 Baseline RR/Spo_2 Complaint with ventilator
1 RR >10 above baseline or 5% ↓ Spo_2 Mild asynchrony with ventilator
2 RR >20 above baseline or 10% ↓ Spo_2 Severe asynchrony with ventilator

5.b PhysiologicII (0-2) =

0 Warm, dry skin
1 Dilated pupils,perspiring, flushing
2 Diaphoretic, pallor
